# Temporal Dynamics of Resting-state Functional Networks and Cognitive Functioning following Systemic Treatment for Breast Cancer

**DOI:** 10.1007/s11682-022-00651-8

**Published:** 2022-06-15

**Authors:** Maryse J. Luijendijk, Biniam M. Bekele, Sanne B. Schagen, Linda Douw, Michiel B. de Ruiter

**Affiliations:** 1 Department of Psychosocial Research and Epidemiology, Netherlands Cancer Institute, Antoni Van Leeuwenhoek Hospital, Amsterdam, The Netherlands; 2 Brain and Cognition Group, Department of Psychology, University of Amsterdam, Amsterdam, The Netherlands; 3 Department of Anatomy and Neurosciences, Amsterdam University Medical Centers, Vrije Universiteit Amsterdam, Amsterdam Neuroscience, Cancer Center Amsterdam, Amsterdam, The Netherlands

**Keywords:** Breast cancer (BC), Cancer-related cognitive impairment (CRCI), Dynamic functional connectivity (dFC), Resting-state fMRI (rsfMRI)

## Abstract

Many women with breast cancer suffer from a decline in memory and executive function, particularly after treatment with chemotherapy. Recent neuroimaging studies suggest that changes in network dynamics are fundamental in decline in these cognitive functions. This has, however, not yet been investigated in breast cancer patients. Using resting state functional magnetic resonance imaging, we prospectively investigated whether changes in dynamic functional connectivity were associated with changes in memory and executive function. We examined 34 breast cancer patients that received chemotherapy, 32 patients that did not receive chemotherapy, and 35 no-cancer controls. All participants were assessed prior to treatment and six months after completion of chemotherapy, or at similar intervals for the other groups. To assess memory and executive function, we used the Hopkins Verbal Learning Test – Immediate Recall and the Trail Making Test B, respectively. Using a sliding window approach, we then evaluated dynamic functional connectivity of resting state networks supporting memory and executive function, i.e. the default mode network and frontoparietal network, respectively. Next, we directly investigated the association between cognitive performance and dynamic functional connectivity. We found no group differences in cognitive performance or connectivity measures. The association between dynamic functional connectivity of the default mode network and memory differed significantly across groups. This was not the case for the frontoparietal network and executive function. This suggests that cancer and chemotherapy alter the role of dynamic functional connectivity in memory function. Further implications of these findings are discussed.

## Introduction

Among women, breast cancer (BC) is the most frequently diagnosed cancer and the leading cause of death worldwide (Sung et al., 2021). With advances in diagnosis and treatment, survival rates have increased, which has led to increased focus on post-treatment side effects (Wefel et al., 2015). A commonly reported side effect is cancer-related cognitive impairment (CRCI). Up to 75% of cancer patients face cognitive decline, particularly after chemotherapy (Ahles & Root, 2018; Mayo et al., 2021). This may persist up to years after treatment (de Ruiter et al., 2011; Koppelmans et al., 2013) and can severely impair quality of life and well-being (Boykoff et al., 2009). Memory and executive function are among the most prominently affected cognitive domains (Ahles & Root, 2018; Mayo et al., 2021).

Several studies demonstrate alterations in functional connectivity of whole-brain networks as measured with resting-state fMRI (rsfMRI) that are associated with reduced cognitive functioning in women with BC (Cheng et al., 2017; Feng et al., 2020; Kesler et al., 2013). Notably, these studies focused on stationary functional connectivity (sFC), averaged over the entire scanning session, based on the assumption that connections within large-scale functional networks would remain stable during this time. However, connectivity strength fluctuates over short time scales and recently it has been argued that these network dynamics are fundamental in supporting cognitive function (Chang & Glover, 2010; Hutchison et al., 2013; Lurie et al., 2020; Sizemore & Bassett, 2018).

Dynamic functional connectivity (dFC) is a measure that reflects the fluctuations in connectivity strength in windows of time sliding over a time series (Chang & Glover, 2010; Hutchison et al., 2013; Lurie et al., 2020). This is hypothesized to promote flexibility of functional networks, reflecting the ability of the brain to adapt quickly and dynamically to fluctuating environmental demands (Cohen, 2018). It allows the brain, for example, to rapidly reconfigure and recruit the brain regions necessary to successfully complete a task at hand. Several previous studies have linked dFC to cognitive functioning (Cohen, 2018; Douw et al., 2015; Douw et al., 2016; Eichenbaum et al., 2021; Kucyi et al., 2018; van Geest et al., 2018; Vidaurre et al., 2021) and dFC seems to outperform sFC in explaining cognitive variance (Eichenbaum et al., 2021; Jia et al., 2014). The precise role of dFC in cognitive function varies depending on brain-state (rest vs. task) and specific resting-state network or cognitive domain (Jia et al., 2014). For instance, higher resting-state dFC of the default mode network (DMN) has been associated with better memory function (Douw et al., 2015; Engels et al., 2018). In contrast, lower resting-state dFC and higher task-state dFC of the frontoparietal network (FPN) have been associated with greater cognitive flexibility (Douw et al., 2016).

Up till now, only one study examined dFC in women with BC, prior to any treatment, and found lower whole-brain dFC that was associated with greater global cognitive dysfunction (Kesler et al., 2017). Another study investigated the effect of chemotherapy on dFC in lung cancer patients and found reduced dFC between frontoparietal areas, again associated with lower cognitive function in general (Hu et al., 2020). For BC patients, however, network- or domain-specific findings have not yet been reported. Further investigating dFC of the DMN and FPN in relation to cognition could help us better understand the mechanisms underlying CRCI.

Taken together, previous research suggests that both cancer treatment and cancer itself can disrupt functional connectivity and there is some preliminary evidence that network dynamics are affected as well. As this is a newly emerging field of research, evidence is scarce. To our knowledge, no other study has examined to what extent BC and adjuvant systemic chemotherapy affect temporal dynamics of functional resting-state networks, nor how this relates to domain-specific CRCI. Therefore, we investigated this within a longitudinal, double-controlled study that combines neuroimaging (rsfMRI) and neuropsychological assessment (see Menning et al., 2015). We examined the temporal dynamics of the DMN and FPN as neural correlates of memory and executive function, respectively. Based on literature on network dynamics (Douw et al., 2015, 2016; Engels et al., 2018; Kucyi et al., 2018; van Geest et al., 2018) we hypothesized that lower and decreasing resting-state dFC of the DMN would be associated with lower and decreasing memory function, while higher and increasing resting-state dFC of the FPN would be associated with lower and decreasing executive function.

## Methods

### Participants

Participants were women with BC who had undergone surgery (mastectomy or lumpectomy) and were scheduled to receive adjuvant systemic treatment consisting of anthracycline-based chemotherapy with or without endocrine treatment (BCC +), women with BC who did not require systemic treatment (BCC-), and age-matched no-cancer controls (NC). Inclusion criteria for eligibility were: female, age under 70 years, and sufficient understanding of the Dutch language. Patients had to have a primary diagnosis of BC, no metastases, no previous malignancies, and no other treatment than surgery at baseline. Age- and IQ-matched NC were recruited via patients and via advertisements for hospital personnel.

### Procedures

Data were collected within a prospective, longitudinal study. The study was approved by the Institutional Review Board of the Netherlands Cancer Institute. Written informed consent was obtained and the study was carried out in accordance with the principles of the Declaration of Helsinki and following institutional guidelines and regulations. The experiment was conducted at the Academic Medical Center of the University of Amsterdam and the Spinoza Centre for Neuroimaging. Baseline data were obtained post-surgery but prior to the start of adjuvant systemic therapy. Follow-up data were obtained on average 6 months after the last cycle of chemotherapy, or at similar intervals for both control groups. The assessment consisted of neuropsychological assessment, completion of questionnaires, and multimodal MRI. The current study focuses on neuropsychological performance in relation to resting-state fMRI. Findings in other modalities have been described elsewhere (e.g., Menning et al., 2018).

### Neuropsychological assessment

The neuropsychological test battery consisted of 10 tests (described in detail elsewhere, Menning et al., 2016). The Hopkins Verbal Learning Test-Revised (HVLT-R) (Benedict et al., 1998) immediate recall and the Trail Making Test B (TMT-B) (Reitan, 1958) were used as measures of memory and executive function, in accordance with the recommendations of the International Cognition and Cancer Task Force (ICCTF) (Wefel et al., 2011). HVLT-R parallel versions were used for both time points. The Dutch version of the National Adult Reading Test (NART) (Schmand et al., 1992) was included at baseline to assess premorbid intelligence. All tests were conducted in Dutch.

### MRI acquisition

MRI data were acquired using a 3.0 Tesla Intera full-body MRI scanner (Amsterdam Medical Center) and a 3.0 Tesla Achieva full-body scanner (Spinoza Centre for Neuroimaging), both using a SENSE 8-channel receiver head coil. Participants were instructed to lie in the scanner with their eyes open, while rsfMRI was acquired based on T2*-weighted gradient echo planar imaging (EPI) of 38 axial slices (voxel size 2.3 × 2.3 × 2.3 mm, interslice gap 0 mm, matrix size 96 × 96, TR = 2.1 s, TE = 25 ms). 180 volumes were acquired, resulting in a total scanning time of 6:18 min. In addition, a T1-weighted three-dimensional magnetization prepared rapid gradient echo (MPRAGE) scan was acquired for spatial normalization.

### MRI preprocessing

rsfMRI was preprocessed using a default pipeline within FEAT (FMRI Expert Analysis Tool) Version 6.00, part of FMRIB Software Library (FSL) (https://fsl.fmrib.ox.ac.uk/fsl/fslwiki), combined with removal of movement artifacts based on independent component analysis (ICA-AROMA; Pruim et al., 2015) (https://github.com/maartenmennes/ICA-AROMA). The following preprocessing-steps were performed: head motion correction using MCFLIRT, co-registration to the skull-stripped T1-weighted structural image using boundary-based registration and spatial normalization to the Montreal Neurologic Institute (MNI) template using FLIRT, segmentation of the T1-weighted image into gray matter, white matter, and cerebrospinal fluid using FAST, regressing out signal from non-gray matter in functional scans, high-pass filtering at 0.01 Hz, and spatial smoothing using an 5-mm full-width half-maximum Gaussian kernel. The functional network was constructed using the Automated Anatomical Labeling (AAL) atlas (Tzourio-Mazoyer et al., 2002). The same 90 cortical and subcortical regions of interest (ROIs) were selected as in prior research. The atlas was first warped to native space. Then, the average time series of each of the 90 regions was extracted by averaging the time series of all voxels within that region. Further processing was performed in Matlab, version 2019a (Mathworks, Natick, MA, USA).

### Dynamic functional connectivity

**Fig. 1:**
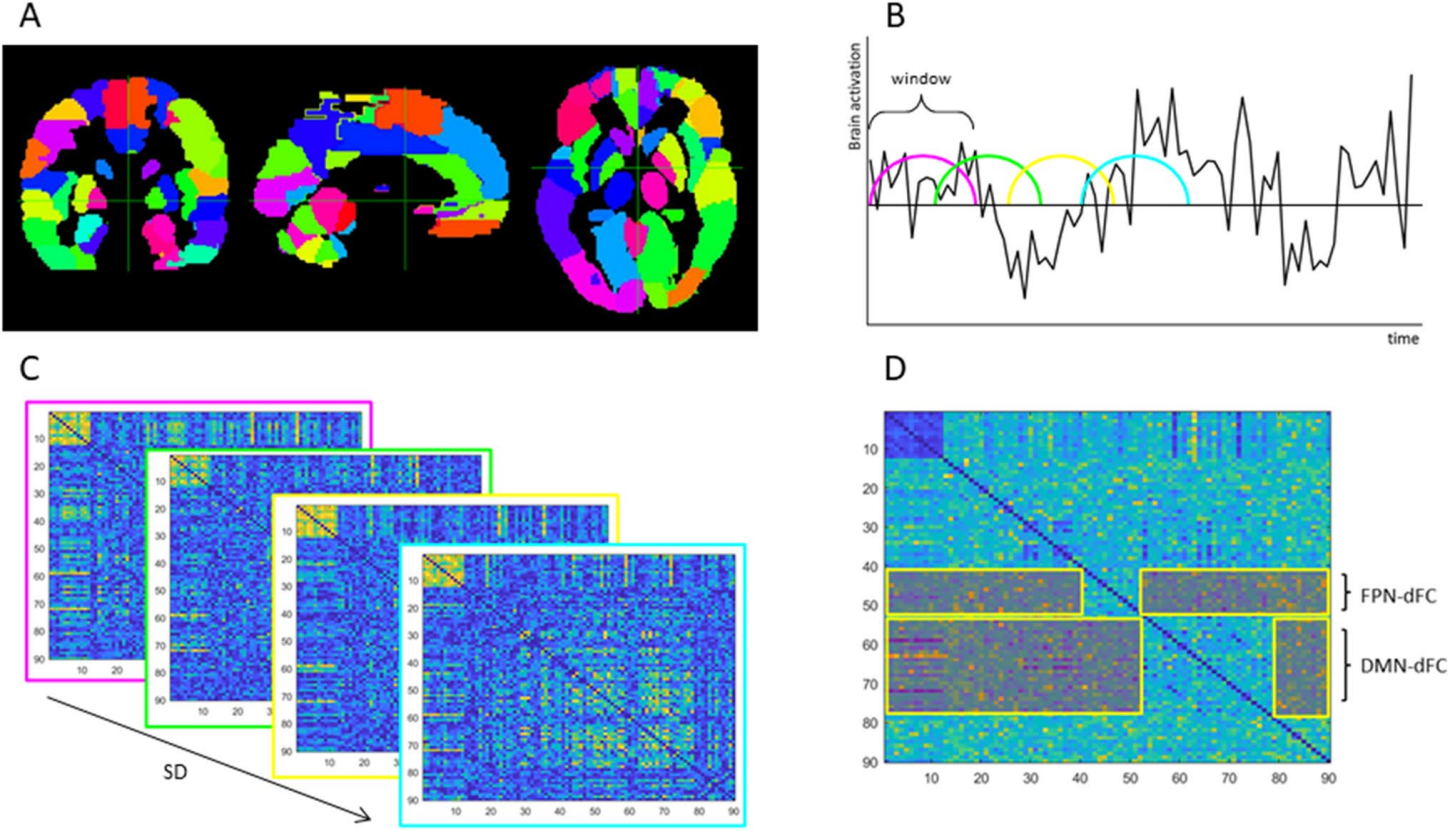
Schematic overview of the sliding window approach. Dynamic functional connectivity (dFC) was calculated using a sliding window approach. A) The cortical surface was parcellated using the Automated Anatomical Labeling (AAL) atlas (Tzourio-Mazoyer et al., 2002) and mean time series were obtained for each cortical and subcortical region. B) For each window, a correlation matrix was computed. C) For each connection, the standard deviation of connectivity was calculated and normalized for its average strength, resulting in a dFC-matrix for each participant. D) Then, the average dFC of all nodes (AAL regions) of the default mode network with the rest of the brain (DMN-dFC) and the average dFC of all regions of the frontoparietal network and the rest of the brain (FPN-dFC) were computed

Using a sliding window approach (Fig. 1), we calculated dFC of the DMN and FPN as measures of variability in functional connectivity of these networks with the rest of the brain. In line with previous studies (Douw et al., 2016; Engels et al., 2018; Leonardi & van de Ville, 2015) we used a window length of 28 volumes (58.8 s) and a shift of 5 volumes (10.5 s), which resulted in a total of 31 windows. For each window, absolute Pearson’s correlation coefficients were computed. Then, we calculated the standard deviation of each connection (i.e., pairwise combination of regions) over windows, which we normalized for the average of that connection to obtain a coefficient of variation that reflects dFC of that particular connection.

The DMN and FPN were defined based on previous resting-state literature (Yeo et al., 2011). Using rsfMRI data from 1000 healthy subjects, Yeo et al. identified seven reproducible resting-state networks. This atlas is used frequently in the field, allowing for optimal comparison between studies. The 78 cortical AAL regions were all assigned to one of these networks and we selected those comprising the DMN and FPN. Out of the dFC matrix we extracted the values of the connections between regions of the DMN and the rest of the brain and the connections between regions of the FPN and the rest of the brain (excluding the within-network connections), which were averaged to obtain single measures of DMN-dFC and FPN-dFC for each participant and measurement.

To verify that variability in connectivity reflected genuine neural dynamics rather than spurious fluctuations due to measurement noise, we also calculated the average whole-brain dFC and compared this against a null model. For each original time series we created 20 randomized copies by using phase randomization on the discrete Fourier transform of the time series (in which sFC and autocorrelation remain preserved) and taking the discrete inverse Fourier transform afterwards (described in detail elsewhere: Hindriks et al., 2016; Prichard & Theiler, 1994). Whole-brain dFC was calculated for each randomized time series, averaged per participant, and then compared to the original whole-brain dFC using a paired t-test.

### Statistical analyses

Statistical analyses were performed using SPSS 25 (IBM, Amonk, NY). To ensure optimal comparability of results, neuropsychological test scores were analyzed using a similar method as in our previous study (Menning et al., 2016): First, raw neuropsychological test scores were converted to standardized z-scores, based on the mean and standard deviation of NC at baseline. Then, predicted follow-up scores were calculated based on baseline performance, age, and IQ using regression coefficients estimated in the NC group. The difference between test scores and predicted scores was calculated to obtain residual scores of follow-up performance (i.e., performance at follow-up corrected for baseline). Univariate differences between groups on cognitive outcomes were analyzed using one-way analyses of variance (ANOVA) on the residual scores. Additionally, we calculated standardized effect sizes (ES) by dividing the mean difference between groups by the pooled standard deviation. ES were computed for the comparisons of BCC + with BCC- and NC.

Because less is known about linearity of change in connectivity measures over time, we did not apply the same method of correcting for baseline for these imaging measures. Rather, we computed delta scores of the difference between the baseline and follow-up measures. Differences in dFC measures between groups and changes over time were examined using repeated measures ANOVAs. We checked for differences in head motion by comparing mean displacements during scanning between groups using one-way ANOVAs.

To examine dFC of the DMN and FPN as neural correlates of CRCI, we performed three-way full-factorial repeated measures analyses of covariance (ANCOVA): one on *memory function* (HVLT-R immediate recall) with the categorical between-subjects variable *group* (BCC + /BCC-/NC), the categorical within-subjects variable *time* (baseline/follow-up), and the continuous within-subjects variable *DMN-dFC change* (ΔDMN-dFC) as independent variables; and one on *executive function* (TMT-B) with *group, time,* and *FPN-dFC change* (ΔFPN-dFC) as independent variables. Confounding effects of age and premorbid IQ were included as covariates in the analyses, considering their well-established relationships with both neuroimaging outcomes and cognitive performance (Damoiseaux et al., 2008; Hearne et al., 2016; Vidal-Piñeiro et al., 2014). In case of significant effects, pairwise comparisons were performed. We used an α threshold of 0.01 to limit the risk of type I errors as a result of multiple testing.

## Results

### Demographics

A total of 52 BCC + , 39 BCC-, and 44 NC were included. Complete datasets, containing both baseline and follow-up rsfMRI, were available for 37 BCC + , 32 BCC-, and 36 NC. Participants with missing rsfMRI scans were excluded. All but 3 BCC + and 1 NC were scanned twice in the same scanner. These four participants were excluded from analysis, resulting in a final sample of 34 BCC + , 32 BCC-, and 35 NC. Standardization of neuropsychological test scores was based on all NC of which neuropsychological data was available at baseline and follow-up, including those of which no rsfMRI data was available, resulting in a norm group of 37 NC. Therefore, our final sample and norm group differed slightly from that in previous reports (e.g., Menning et al., 2016).

Patient characteristics are presented in Table 1. No significant differences in age, premorbid IQ, or education level were found between groups. After chemotherapy, all but one BCC + became post-menopausal. In the same interval, no NC and three BCC- became menopausal.

**Table 1:** Demographics

	BCC + (*N* = 34)	BCC- (*N* = 32)	NC (*N* = 35)	*p*
Age	49.2 (± 9.3)	50.8 (± 7.1)	49.7 (± 10.3)	0.743
IQ	101 (± 13)	105 (± 13)	107 (± 13)	0.119
Education (n(%))				0.424
low	0 (0%)	1 (3.1%)	0 (0%)	
middle	3 (8.8%)	4 (12.5%)	0 (0%)	
high	31 (91.1%)	27 (84.4%)	35 (100%)	
Time since BL (days)	333 (± 62)	341 (± 43)	365 (± 63)	0.057
Time since CHT (days)	192 (± 109)	-	-	
Post-menopausal (n(%))				
BL	14 (41.2%)	15 (46.9%)	18 (51.4%)	0.683
FU	33 (97.1%)	18 (56.3%)	18 (51.4%)	< 0.001 *

Values indicate mean ± SD unless indicated otherwise. BCC +  = breast cancer patients treated with chemotherapy; BCC- = breast cancer patients not treated with chemotherapy; NC = no-cancer controls. Age = age at baseline. IQ = premorbid intelligence as estimated with the Dutch version of the National Adult Reading Test (NART). Education = summarized in three groups: low = primary school; middle = secondary school; high = university of applied sciences or university degree. Time since BL = time between baseline (BL) and follow-up (FU). Time since CHT = time since last cycle of chemotherapy.

* significant difference between groups at *p* < 0.01 (corrected).

### Cognitive function

Average standardized test scores from neuropsychological assessment and standardized effect sizes of the comparisons of BCC + vs. BCC- and BCC + vs. NC are presented in Table 2. Univariate one-way ANOVAs showed no significant differences between groups on the test indices. Notably, the difference between groups on the HVLT-R immediate recall approached significance (*p* = 0.057), performance being slightly better for BCC- compared to the other two groups. Comparing BCC + to BCC-, a small-to-moderate effect size was found for HVLT-R immediate recall (*ES* = -0.47) with BCC + performing worse.

**Table 2:** Cognitive performance

	BCC + (*N* = 34)	BCC- (*N* = 32)	(ES)	NC (*N* = 35)	(ES)	*p*
Executive function						
TMT-B	-1.87 (1.24)	-0.12 (1.01)	0.17	0.04 (0.73)	0.23	0.627
Verbal memory						
HVLT-R immediate recall	-0.07 (0.89)	0.44 (0.91)	-0.47	-0.01 (0.97)	-0.06	0.057

Values indicate z-scores (mean ± SD) at follow-up corrected for baseline. Scores are standardized based on NC (*N* = 37) performance at baseline. Lower scores indicate worse performance. BCC +  = breast cancer patients treated with chemotherapy; BCC- = breast cancer patients not treated with chemotherapy; NC = no-cancer controls; TMT-B = Trail Making Test B; HVLT-R = Hopkins Verbal Learning Test – Revised; ES = Standardized effect size for the post-hoc comparisons BCC + vs. BCC- and BCC + vs. NC. No significant group differences were found.

### Dynamic functional connectivity

**Fig. 2:**
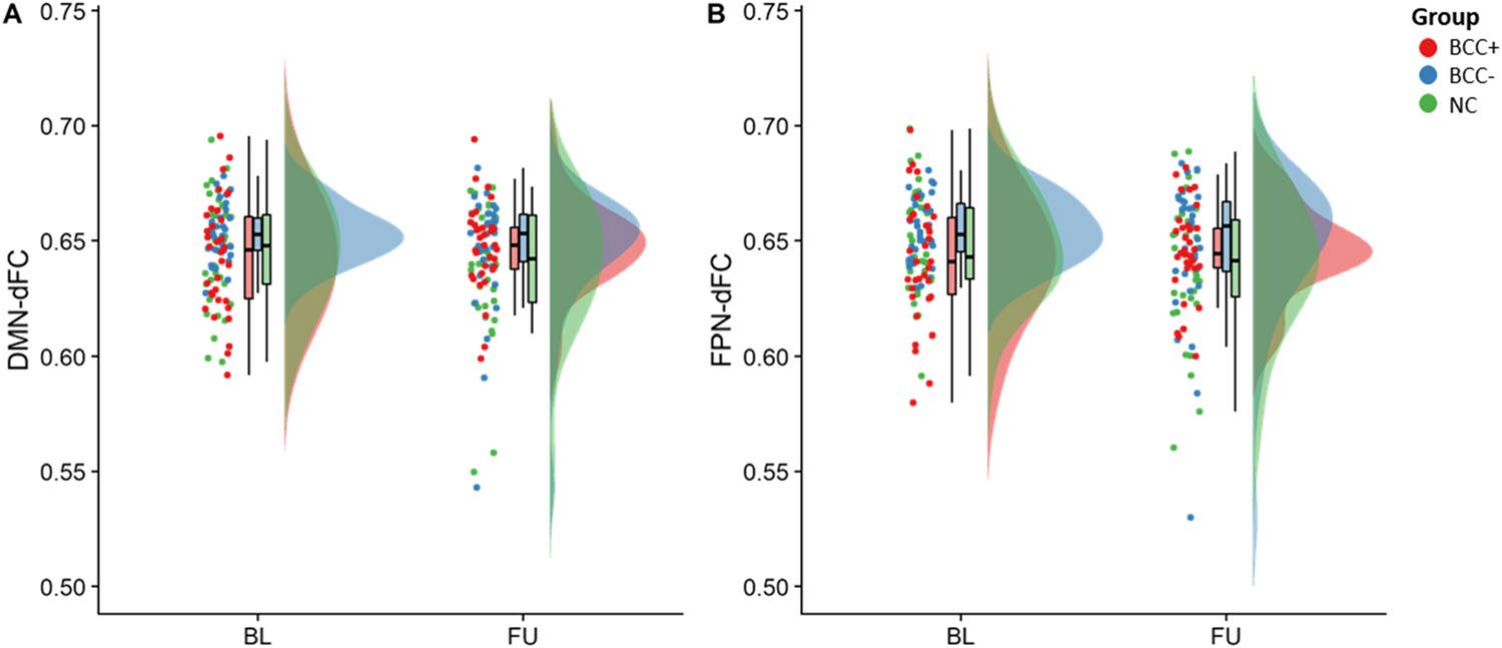
Dynamic functional connectivity. Distributions of dynamic connectivity of the default mode network (DMN-dFC, A) and frontoparietal network (FPN-dFC, B) are visualized at baseline (BL) and follow-up (FU) separately for women with BC treated with chemotherapy (BCC +), not treated with chemotherapy (BCC-), and no-cancer controls (NC). No statistically significant differences were found between groups or over time

Head motion during scanning did not differ significantly between groups (*p* > 0.121), and mean displacements were small (mean absolute displacement: 0.2502 ± 0.1479 mm). Whole-brain dFC was significantly different from that calculated from the randomized time series (*t*(100) = 2.583, *p* = 0.011), suggesting variability reflects brain dynamics rather than measurement noise. Mixed ANOVAs showed no significant differences in DMN-dFC or FPN-dFC between groups or over time (*p* > 0.151) (Fig. 2).

### DMN-dFC and memory function

**Fig. 3:**
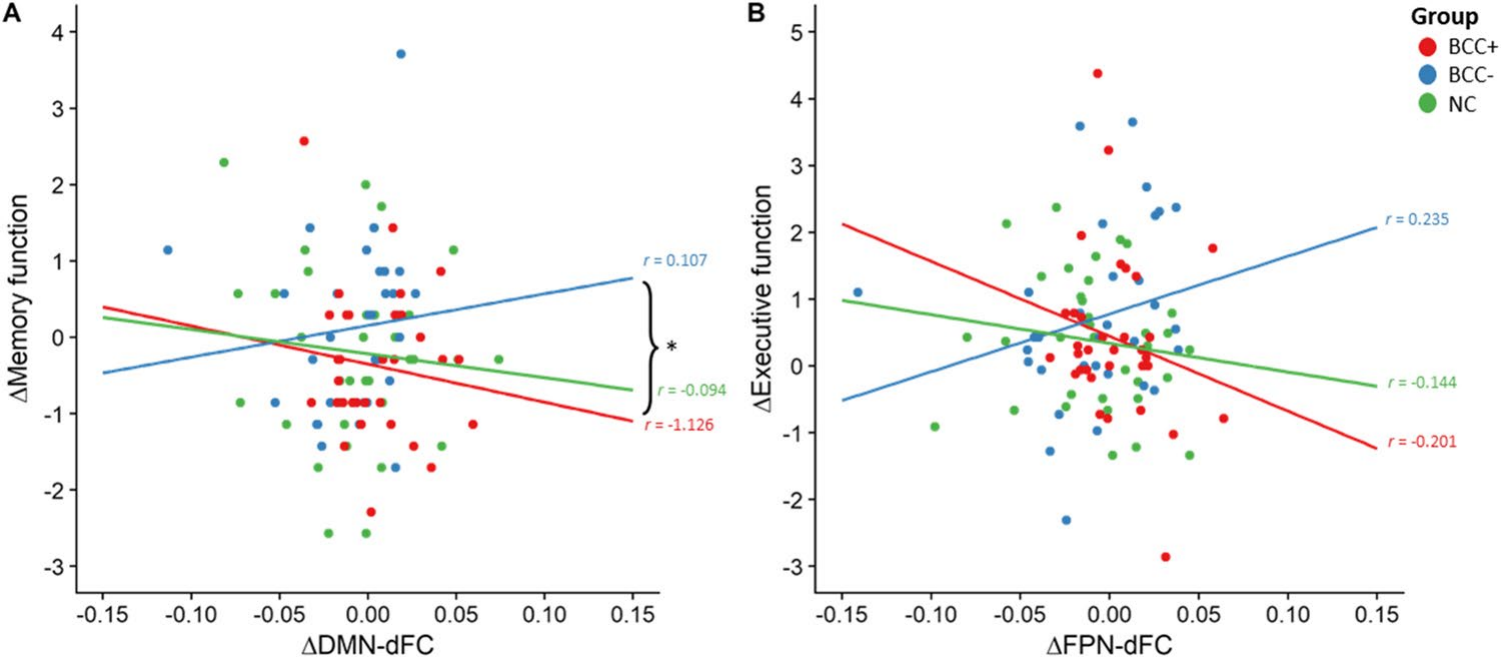
Associations between dynamic functional connectivity and cognition. Delta scores of change in dynamic connectivity of the default mode network and frontoparietal network and their associations with changes in memory and executive function are visualized separately for women with BC treated with chemotherapy (BCC +), not treated with chemotherapy (BCC-), and no-cancer controls (NC). A) The direction of the association between dynamic functional connectivity of the default mode network (DMN-dFC) and memory function (HVLT-R immediate recall) was significantly different between groups (*p* = 0.003), with a positive association for BCC-, that was reversed for BCC + and NC. B) No significant association between dynamic functional connectivity of the frontoparietal network (FPN-dFC) and executive function (TMT-B) was found. * Significant difference at *p* < 0.01 (corrected)

In Fig. 3A the association between DMN-dFC and memory is visualized. A three-way full-factorial repeated measures ANCOVA on memory function showed a significant *group*-by-Δ*DMN-dFC* interaction (*F*(2,92) = 6.192, *p* = 0.003) and a main effect of *time* (*F*(1,92) = 4.297, *p* = 0.041), with a decrease in performance at follow-up (*M* = 0.102 ± 1.129) compared to baseline (*M* = 0.254 ± 1.146). There was no significant main effect of *group* (*F*(2,92) = 0.772, *p* = 0.465) or Δ*DMN-dFC* (*F*(1,92) = 2.362, *p* = 0.128), nor were any of the other interactions significant (*p* > 0.117). While the covariate age was significantly related to memory function (*F*(1,92) = 3.982, *p* = 0.049), IQ only trended towards significance (*F*(1,92) = 3.006, *p* = 0.086). Only the *group*-by-Δ*DMN-dFC* interaction survived correction for multiple comparisons. Figure 3A visualizes the inhomogeneity of regression slopes across groups: whereas in the BCC- group an increase in DMN-dFC was associated with an increase in memory function at follow-up, the direction of this relationship was inversed in the BCC + and NC group.

### FPN-dFC and executive function

In Fig. 3B the association between FPN-dFC and executive function is visualized. A three-way full-factorial repeated measures ANCOVA on executive function showed no significant main effect of *time* (*F*(1,92) = 1.538, *p* = 0.218), *group* (*F*(2,92) = 2.613, *p* = 0.079), or Δ*FPN-dFC* (*F*(1,92) = 1.228, *p* = 0.271), nor were any of the interaction effects significant (*p* > 0.113). Age was significantly related to executive function (*F*(1,92) = 11.537, *p* = 0.001). Again, IQ only trended towards significance (*F*(1,92) = 3.370, *p* = 0.070).

## Discussion

The current study investigated the effect of adjuvant systemic therapy on dynamic functional connectivity of the default mode network (DMN-dFC) and frontoparietal network (FPN-dFC) in women with BC, as neural correlates of memory and executive function, respectively. While we did not find a relation between FPN-dFC and executive function, for DMN-dFC the direction of the relationship with memory function appeared to differ across groups. This is suggestive of a potential, albeit complex, role of DMN-dFC in memory function. However, due to absence of group differences or changes over time in both cognitive and neuroimaging outcomes, our findings can neither confirm nor reject whether alterations in DMN-dFC or FPN-dFC underlie decline in memory or executive function.

Due to its neurotoxicity, chemotherapeutic treatment is thought to cause alterations in functional connectivity of large-scale whole-brain networks. We hypothesized that their temporal dynamics would be affected as well and that this would be related to cognitive function. Unlike the only other study that looked into functional network dynamics in women with BC (Kesler et al., 2017), we did not observe decreased dFC prior to treatment in the BC groups. Neither did we observe changes in dFC following chemotherapy, while Hu et al. (2020) did in lung cancer patients. However, our results do show a relation between DMN-dFC and memory function. This seems to be in line with other studies applying a sliding-window approach to assess dFC of subnetworks, although in different patient groups, where despite a lack of group differences a positive association between (DMN-)dFC and memory was found (Engels et al., 2018; van Geest et al., 2018).

Remarkably, our results show that the relation of DMN-dFC with memory function is different between groups: whereas for BCC- we found a positive relationship, meaning that lower dFC was indeed associated with lower memory function, the direction of this relation was reversed for BCC + and NC. This finding is suggestive of a differential role of DMN-dFC for memory function within different populations. Interestingly, previous literature has found associations between dFC and memory function in both directions as well (Douw et al., 2015; van Geest et al., 2018). These findings hint towards a complex interplay between different subnetworks which comprise the DMN that could be important for memory function. Differences between subnetworks have been found before, with decreased dFC in some DMN-subnetworks but increased dFC in others (de Lacy et al., 2017). Differences can even be found at the single node level, where both low-flexible and high-flexible nodes contribute to cognitive performance (Bassett et al., 2011). Moreover, flexibility seems to be modulated by the stage of learning, with higher levels of flexibility in early stages and lower flexibility as performance stabilizes (Bassett et al., 2011; Newell et al., 2009). Our findings combined stress the importance to further investigate the complex role of (DMN-)dFC in learning and memory.

On the contrary, we could not establish an association between resting-state FPN-dFC and executive function, although associations were found before (Boon et al., 2020; Douw et al., 2016). These inconsistencies could be explained by differences in assessing FPN-dFC. Alternatively, it could be that our task required a different executive process that does not rely on FPN-dFC as we defined it. Furthermore, FPN-subnetwork analysis and the interplay between resting-state and task-state might again be more informative, as it has been suggested that different FPN connectivity patterns are related to different cognitive task states (Cole et al., 2013) and dynamic changes occur when moving between these different states (Denkova et al., 2019; Lurie et al., 2020).

Our study knows several limitations. First, our sample shows a relatively low incidence of cognitive decline compared to previous studies (Ahles & Root, 2018; Mayo et al., 2021). A noticeable characteristic of our sample—presumably due to participation bias—is that it consisted of women that are relatively young of age and highly educated, which might protect against brain damage and subsequent cognitive decline (Ahles et al., 2010). Due to the lack of cognitive decline, the question whether changes in dFC underlie CRCI remains unanswered. Second, although our sample sizes are comparable to that of previous neuroimaging studies in this field, we might still have insufficient power to detect the subtle effects of chemotherapy on both cognitive and neuroimaging measures, especially considering our complex statistical analyses. Notably, we did observe a small-to-moderate effect size for worse memory performance for BCC + compared to BCC-, resembling the negatively deviating pattern of test scores for BCC + previously observed (Menning et al., 2016). Third, because of selection bias participants might differ in their motivation and reason to participate. Moreover, because one BC group required intense chemotherapy while the other did not, their disease course and stage of emotional processing might have been different. These psychological factors could have influenced the results and explain why the BCC- group seemed to improve in memory function, as well as on other neuropsychological outcomes (Menning et al., 2016), and reported reduced fatigue (Bekele et al., 2021). Fourth, baseline assessment occurred post-surgery so we are unable to rule out possible confounding effects of surgery or anesthesia on the baseline assessment in the BC groups. Fifth, whereas equivalence of the American HVLT-R parallel versions has been demonstrated (Benedict et al., 1998), normative data for the Dutch versions are lacking and their equivalence remains to be investigated. Possibly, differences in their difficulty might have influenced the results.

Lastly, the numerous methodological choices one has to make when analyzing neuroimaging data can have a considerable impact on data quality and results: for instance, fMRI preprocessing steps and their order (Gargouri et al., 2018), the choice of parcellation atlas (Power et al., 2011; Zalesky et al., 2010), the definition of resting-state networks, and how to deal with confounding effects of physiological noise and motion artifacts (Power et al., 2012; Wang et al., 2011). Similarly, there is no known optimal method of assessing network dynamics yet (Lurie et al., 2020). Although we used a relatively simple sliding-window approach, still several choices had to be made that might influence results, such as the choice of window length and shift or the measure to assess dFC (Preti et al., 2017) and whether to take into account negative correlations (Shehzad et al., 2009). Because we were interested in whole-brain network functioning, we chose to compute dFC by averaging across all connections of a network. While this might result in lower sensitivity to detect local changes in dFC, this greatly reduces the potential influence of noise. Moreover, all currently used methods and choices are in line with what has been used often in this field, enabling optimal comparison of results. Concluding, since the field of network dynamics emerged only very recently, there are no best practices yet. It is therefore important to investigate which methods yield the most valid and reliable results.

## Conclusions

To our knowledge, this is the first study that examined the influence of adjuvant systemic therapy on temporal dynamics of resting-state networks in the context of CRCI using a longitudinal, double-controlled design. Although we could not identify the neural correlates of CRCI, our study highlights the complex role of network dynamics for cognitive function and the importance to further investigate this in both patient and healthy populations.

## Data Availability

Data generated and/or analyzed during the study are available from the corresponding author on reasonable request.

## Code availability

Code for analysis is available upon reasonable request.
